# Clinical Management and Risk Stratification of Retroperitoneal Sarcoma with a Focus on Locally Recurrent Disease

**DOI:** 10.1007/s11864-026-01418-9

**Published:** 2026-11-03

**Authors:** Ryan D. Watkins, Lee D. Cranmer, Sagus Sampath, Adam G. Rock, Bridget N. Fahy, William W. Tseng

**Affiliations:** 1 Department of Surgery, Division of Surgical Oncology, City of Hope National Medical Center, Duarte, 91010 USA; 2 Department of Medical Oncology and Therapeutics Research, City of Hope National Medical Center, Duarte, USA; 3 Department of Radiation Oncology, City of Hope National Medical Center, Duarte, USA

**Keywords:** Risk-stratification, Recurrent, Retroperitoneal sarcoma

## Abstract

Retroperitoneal sarcomas are a group of rare malignancies comprised of several histological types, each carrying a unique risk of local recurrence and metastatic potential. In primary disease, surgery remains the mainstay of treatment and is the best opportunity for cure. In recurrent disease, treatment algorithms are less well defined. Surgery, radiation, systemic therapy, and in select cases, observation can be considered for management of recurrent disease. Histologic type drives a significant portion of clinical decision-making but incorporating all relevant clinicopathological data is important. To guide selection and timing of treatment, patients should undergo risk stratification. Nomograms and risk calculators are available to facilitate this. Experience and clinical judgement remain paramount. In the future, deeper molecular and radiologic characterization may further optimize decision-making in these challenging cases.

## Introduction

Sarcomas encompass a group of cancers that arise from the bones and soft tissues of the body. The putative tissue of origin defines each sarcoma histologic type; however, for certain sarcomas, this is not well defined and increasingly, unique genomic aberrations are used to define sarcoma types [1]. Collectively, soft tissue sarcomas (STS) are rare and represent about 1% of all solid malignancies in adults [2]. By anatomic location, 15–20% of STS occur in the retroperitoneum [3]. The predominant histologic types of retroperitoneal sarcomas (RPS) include well-differentiated liposarcoma (WDLPS), dedifferentiated liposarcoma (DDLPS), leiomyosarcoma (LMS), malignant peripheral nerve sheath tumor (MPSNT), undifferentiated pleomorphic sarcoma (UPS), and solitary fibrous tumor (SFT). Each RPS type has unique pathologic and clinical characteristics including patterns of recurrence [4–7], which is summarized in Table 1.

**Table 1:** Retroperitoneal sarcoma histologic types and their clinical characteristics

Histologic type	Tissue of origin	Genomic Aberrations	Local Recurrence Risk (5-year incidence, %)	Metastatic Potential (5-year incidence, %)	5-Year OS
Well differentiated Liposarcoma	Adipose tissue	Chromosome 12q amplification; MDM2/CDK4 amplification	Moderate 20–40%	None 0%	87.4%
Dedifferentiated Liposarcoma: Grade 2 (G2) and Grade 3 (G3)	Adipose tissue	Chromosome 12q amplification; MDM2/CDK4 amplification	Moderate 44% (G2) 33% (G3)	Low-Moderate 9.5% (G2) 44% (G3)	54% (G2) 40.8% (G3)
Leiomyosarcoma	Smooth muscle	Multiple	Low 5.8%	High 55%	57.6%
Malignant peripheral nerve sheath tumor	Schwann cells	NF1 loss	Moderate 20%	Low 12.5%	66.7%
Solitary Fibrous Tumor	Mesenchymal cells	NAB2:STAT6 fusion	Low 4.2%	Low 17%	85%
Undifferentiated pleomorphic sarcoma	Fibroblastic/Mesenchymal cells	Complex. No characteristic alteration.	-	-	-

[5–7]

This article will provide an overview of current management and risk stratification for RPS, beginning with primary disease and then focusing on locally recurrent disease.

## Management of Primary Disease

Surgery is the mainstay of treatment for primary RPS. Treatment center volume has been established as a key prognostic factor in primary disease treatment, with improved outcomes at high-volume centers [8–10]. In fact, a minimum threshold volume of 13 RPS cases per year has been proposed as defining “high-volume,” with some centers seeing more than 20 cases per year [11, 12]. Among high-volume centers, overall survival (OS) over the prior 15 years improved and rates of incomplete (R2) resection decreased. However, incidences of local recurrence and distant metastasis have not changed [13]. Despite improved surgical technique for primary RPS, local recurrence continues to affect at least 35–45% of patients at 10 years with no apparent plateau thereafter [5, 6].

While the technical details of surgery are beyond the scope of this discussion, it is universally accepted that the goal of surgery is to achieve, at a minimum, complete (R0/R1) resection while avoiding tumor rupture. Some high-volume European sarcoma centers advocate for an extended or compartmental resection, systematically removing the tumor *en bloc* with adjacent organs and structures when feasible and safe, even without obvious involvement. This approach seeks to further optimize soft tissue “margins” and significantly reduces risk of local recurrence versus simple complete resection [14, 15]. Other sarcoma centers have challenged this approach, with more recent data showing no difference in outcomes when a more selective approach to *en bloc* organ resection, limited to obvious involvement, is employed [16, 17]. Current consensus suggests a histology-tailored approach with more radical resection in appropriately selected patients for those tumor types with high local recurrence risk (WDLPS, DDLPS); less radical approaches can be targeted to those with lesser risk of local recurrence and high metastatic potential (e.g., LMS).

In primary disease, treatment paradigms for primary RPS involving neoadjuvant radiation and chemotherapy are evolving. The EORTC-6292 STRASS trial and the companion off-trial STREXIT study examined the benefit of preoperative radiation therapy followed by surgery versus surgery alone. Overall, there was no significant benefit conferred by preoperative radiation therapy, versus surgery alone, although there was approximately a 50% reduction in local failure rate with radiation. A *post hoc* subgroup analysis demonstrated that patients with WDLPS and grade 1–2 DDLPS had an approximately 10% decrease in incidence of the composite abdominal recurrence outcome [18].

There continues to be heterogeneity across the world in utilization of preoperative radiotherapy; in a recent pre/post STRASS multicenter analysis, only 14% of patients received preoperative radiotherapy [19]. STRASS 2 is an ongoing multi-national prospective randomized control trial to evaluate the efficacy of neoadjuvant chemotherapy followed by surgery compared to surgery alone in patients with high risk of distant metastasis (grade 3 DDLPS and LMS) [20]. Study accrual is expected to continue until early 2028. Results of this trial may further shape the management of primary RPS.

## Risk Stratification in Primary Disease

Stratifying for the risk of local and distant recurrence in retroperitoneal sarcoma is the foundation from which all management decisions stem. Factors to consider when risk stratifying can be divided into biological factors, inherent to the patient and the tumor, versus treatment-related factors. These factors are summarized in Table 2.

**Table 2:** Risk factors utilized in risk stratification of primary and recurrent retroperitoneal sarcoma

Clinical Setting	Primary RPS	First recurrence	Second Recurrence
Biological Factors	Age Histologic type Histologic grade Multifocality Tumor size	Age Histologic type Histologic grade Multifocality Disease-free interval Tumor growth rate	Disease-free interval
Technical /Treatment Related Factors	Complete resection Multi-organ resection Tumor rupture	Complete resection Multi-organ resection for primary tumor Chemotherapy or radiation given	Surgery performed

### Biological Factors

Multiple studies have examined patient and tumor factors that significantly impact disease-free survival (DFS) and OS after surgery. Across these studies, the following factors have consistently been found to be important in stratifying patients with primary RPS: patient age, tumor size, multifocality, histologic type, and grade [4, 6, 21–25].

Biological factors are not subject to alteration in and of themselves. They do form the basis of prognostication and decision-making regarding interventions. Their accurate measurement is thus critical. In some cases, such as size determination through cross-sectional imaging, exact and precise determinations can be made. In other circumstances, classification and measurement is more nuanced.

Histologic classification and assessment is one such critical factor that can be very operator dependent. These classifications should be carefully considered and have a low threshold for confirmatory expert review. Frequently, diagnoses are modified when reviewed by an expert sarcoma pathologist [26, 27]. Table 1 highlights the distinct differences in risk of local recurrence and metastatic potential, largely attributable to the histologic type of RPS. The importance of expert review should therefore not be underestimated. Changes in the pathologic assessment, even seemingly minor (e.g., grade 2 versus 3 for liposarcoma), can directly impact management decisions.

### Treatment Factors

Treatment factors can be divided into decisions regarding what treatment to undertake, presumably directed by risk and underlying biology, and the implementation of those decisions. Appropriate decisions in each circumstance may be subject to unexpected adverse outcomes (for example, unanticipated tumor rupture). In other cases, the treatment decision may be less than optimal, such as when a patient requires emergency surgery for an unanticipated abdominal complication, preventing the optimal planned surgical treatment from being undertaken.

Not surprisingly, given its centrality in RPS treatment, the quality of surgery performed impacts patient outcome. The completeness of resection (R2 vs. R0/R1), tumor rupture, and whether multi-organ resection was done all contribute to the overall risk of recurrence [4, 6, 28, 29]. The performance of specific organ resection, including major vessels, does not specifically contribute to risk of disease recurrence, but may significantly alter future treatment planning and intervention, both surgical and otherwise (i.e. chronic renal insufficiency from nephrectomy impacting chemotherapy selection) [30].

These biological and treatment factors have been incorporated into nomograms that are practical decision-making tools to stratify patients by disease-specific survival (DSS)and OS. Two such nomograms used frequently to guide primary RPS treatment include the Memorial Sloan Kettering Cancer Center (MSKCC) nomogram and Sarculator [6, 31]. Both were generated from large volume and in the latter, multicenter retrospective analyses. The MSKCC nomogram additionally provides estimates of local and metastatic recurrence risk [6]. The Sarculator, developed with patient databases from mostly European sarcoma centers, has also recently been validated using a United State National Cancer Database (NCDB) cohort [10]. It is important to note that both nomograms were designed in patients undergoing upfront surgical resection. Utilization of these nomograms for patients who underwent neoadjuvant therapy may overestimate risk [32]. The Sarculator nomogram also provides information regarding outcomes when comparing treatment at high- and low-volume centers, highlighting the advantages of treatment at the former, if possible.

## Management of Recurrent Disease

In comparison to primary disease, recurrent RPS presents added challenges to clinical management. Logistically, patients with recurrent disease may have been cared for at another center and as such, the details of the prior evaluation and treatment may not be readily available. Confidence in the pathologic diagnosis is again critical: if this is at all in doubt, a second review of archived tissue or repeat biopsy may be warranted, especially if this data could change management (e.g., new presence of DD in the context of previous WD LPS).

While surgery still plays a significant role in the management of recurrent RPS, treatment is frequently multimodal; as such, multidisciplinary discussion is especially important. Treatment rendered previously, whether surgical or non-surgical (e.g., radiation), will impact the selection of therapeutic options available for the current recurrence. As in the primary disease, thorough patient evaluation, including existing co-morbidities and performance status, is necessary to estimate tolerance of planned treatment (e.g., reoperative surgery).

Expert consensus guidelines exist for the management of recurrent RPS [33, 34]. However, there is a severe paucity of data in this context versus what is available for primary disease. Nonetheless, for the following discussion, we will examine the key studies for recurrent disease, specifically focused on non-metastatic, locally recurrent disease. The topic of management of metastatic recurrence is beyond the scope of this review. It is also important to recognize that among the different RPS histologic types, local recurrence is predominantly seen in LPS (see Table 1) and is in fact the major clinical issue for both WD and DD LPS patients.

### Observation

While seemingly counter-intuitive, adoption of an expectant management strategy of initial close observation with serial cross-sectional imaging may be a reasonable option in recurrent disease that is exclusively WDLPS [29, 33, 34]. In contrast to DD, WDLPS can display indolent growth and lacks metastatic potential. Upfront observation, allowing assessment of growth kinetics, can be very informative. In fact, need for early re-operation within 12 months of primary surgery has been associated with worse DFS and OS and might provide a rationale for considering other treatments in conjunction, such as radiation or systemic therapy [35].

### Surgery

For most patients with recurrent RPS, surgery remains the mainstay of treatment. Patients who undergo complete resection have significantly better outcomes compared to those who are unresectable or undergo incomplete resection [25, 34, 36–39]. In carefully selected patients, incomplete resection may still have benefit (e.g., symptom control) [40]. One must recognize that in comparison to primary disease, any abdominal operation for recurrence is likely to be more technically challenging. Adhesions from prior surgery and, in some patients, prior radiation therapy can obscure dissection planes. The anatomic relationships of organs and critical structures (e.g., major vessels) are frequently altered. While one multicenter study did not find significantly increased morbidity or mortality in recurrent disease versus historical data for primary disease [41], the risks of reoperative surgery (e.g., bowel injury or fistulization) are well recognized. Multiple recurrences are especially common in WDLPS and DDLPS. The technical challenges of reoperative surgery are in general amplified with each subsequent surgery.

### Radiation

If radiation therapy has not been given previously (e.g., in the primary setting), this therapeutic option should be considered [34]. Several studies demonstrate that external beam radiation therapy, most commonly intensity-modulated radiation therapy, sometimes combined with intra-operative radiation therapy, has a reasonable safety profile and is feasible in the setting of RPS recurrence [42–44]. Additional applications of radiation therapy include brachytherapy. Interestingly, in one study, definitive radiation in the setting of unresectable RPS achieved local control up to 12 months in 10/14 patients and thus may be considered in lieu of surgery for select patients [45]. In patients having received prior radiation, re-treatment selectively to the posterior portion of the tumor may be considered. This has been shown in a pilot trial to be feasible and does not adversely affect surgical outcomes [46].

### Systemic Therapy

Systemic therapy can be considered for two reasons: first, to mitigate the potential for metastatic disease for those tumors at risk and second, to attempt to downsize the tumor to facilitate resection. As in primary disease, the histologic type will guide risk assessment for distant metastasis (see Table 1). In leiomyosarcoma, distant metastasis is the dominant mode of treatment failure, with local recurrence being uncommon. For DDLPS, the relative impact of local and distant recurrence varies with grade, but local recurrence is the major mode of treatment failure, with distant recurrence becoming important in those with high-grade G3 disease. Thus, objectives for using systemic therapy will vary depending on the underlying histology.

In DDLPS and leiomyosarcoma, upfront systemic therapy can be considered, although this approach in recurrent RPS has not yet been formally studied and is not universally accepted. Tumor downsizing with cytotoxic chemotherapy, when extrapolated from primary disease, occurs with partial response rates approximately 20% [47–49]. Hypothetically, tumor response may be a surrogate marker for microscopic disease control systemically, but to date this benefit also has not been demonstrated. As additional targeted therapies with more favorable toxicity profiles become available for RPS (e.g., CDK4-inhibitors in LPS), treatment paradigms may shift to increased usage of these therapies over cytotoxic regimens. There is a clear need for prospective, randomized trials to define the role of systemic agents in this setting.

#### Management of Second and Subsequent Recurrences

Second and subsequent recurrences in RPS are even less well studied. In one multicenter study of 567 patients that underwent complete resection of their first local recurrence, 70% of patients developed a second recurrence [50]. Utilization of non-surgical therapies for this group of patients was quite heterogenous. Radiation therapy was used in the minority of patients for the first recurrence (12.7%) and with a similar frequency for the second recurrence (14.2%). By contrast, chemotherapy utilization markedly increased for patients with second local recurrence compared to the first recurrence (36.8% from 16.7%). For the second recurrence, 62% of patients underwent resection. Patients deemed eligible for surgery, regardless of the type of resection (R0/R1 vs. R2) undertaken, demonstrated markedly improved median OS of 77 months compared to 18 months for those deemed ineligible for surgery. Among those undergoing complete resection of their second recurrence, 65.6% had a third local recurrence within 5 years versus 9.2% with distant metastasis, highlighting local control as the dominant failure mode.

## Risk Stratification in Recurrent Disease

Given the overall paucity of evidence to guide treatment in recurrent RPS, risk stratification is important to guide clinical decision-making. Risk stratification in recurrent RPS focuses on recognition of histologic type and several key factors including pattern of recurrence, extent of disease, and the kinetics of tumor growth. These factors together reveal disease biology, which is arguably most critical to understand for each patient with recurrent disease [51].

Outcomes of recurrent RPS, like primary RPS, are dictated by the histologic type (Table 1). In recurrent LPS, change in histologic subtype (WD vs. DD) and grade may also occur and have prognostic implications. A previously WD or DD tumor may not necessarily remain the same subtype at recurrence. In a multicenter study, favorable outcomes were observed in patients who have a WDLPS recurrence after having DDLPS and worse outcomes for the opposite (DD after initial WD) [41]. Thus, it is important to have an accurate histologic diagnosis for the current disease burden, obtained by expert radiologic assessment with a low threshold to obtain core needle biopsy.

The pattern of disease recurrence based on high-quality cross-sectional imaging should distinguish between local versus metastatic recurrence or a combination of both. In determining the extent of the disease burden, particularly in LPS, there should be consideration that local recurrence can potentially occur throughout the entire RP, intraabdominal and pelvic space. As such, the term “locoregional” recurrence may be appropriate. The specific location of the recurrence (relative to the primary or previous tumor) and extent of organ involvement and residual disease resulting from an incomplete resection of the primary tumor should be distinguished from and de novo recurrence. The number of tumors seen is also important: multifocal disease − 2 or more discontiguous tumors not arising from visceral organs - is much more common in recurrent versus primary disease: 50–65% vs. 9–20% [5, 22, 25, 52]. This can be iatrogenic if the previous surgery was incomplete or piecemeal or potentially, a natural disease process. Multifocality as a prognostic factor is independently associated with worse outcomes [39]. As such, careful radiologic examination is needed with the awareness that in some cases, multifocality can only be definitively determined intraoperatively (e.g., 2 adjacent tumors vs. 1 large, multilobulated tumor).

Temporal trends or the kinetics of tumor growth should be studied for each patient with recurrent RPS. This should first include determination of the disease-free interval (DFI), defined as time to recurrence from the initial primary tumor resection or, in the case of multiple recurrences, from the last resection. Several studies in recurrent RPS have identified that longer DFI is associated with better overall outcomes [50, 53]. This was confirmed in an additional retrospective analysis which showed that a cutoff DFI < 1 year was associated with worse OS (HR 3.64) [35]. In fact, patients with an even shorter DFI < 6 months had remarkably worse DSS (HR 2.18) but not OS [54]. As expected, DFI typically shortens with each subsequent recurrence [50, 55]. Apart from DFI, the growth rate of the recurrent tumor itself is also associated with worse outcomes [56]. In an earlier single-center study, this was defined as the size of the tumor in centimeters divided by the DFI in months from primary tumor resection. A growth rate > 0.9 cm / month was associated with worse outcomes.

The factors highlighted above and others have been incorporated into the Sarculator [31, 39]. The current update to Sarculator estimates DFS and OS from the second surgery for the first recurrence. This determination utilizes age, Fédération Nationale des Centres de Lutte Contre le Cancer (FNCLCC) grade, histology, multifocality, as well as treatment related factors: completeness of resection, chemotherapy or radiotherapy for the primary tumor, and number of resected organs at the first surgery [39]. This risk stratification is done only after surgery. Importantly while these nomograms can be useful, they should not replace experience and sound clinical judgement.

Beyond the first recurrence, when evaluating patients with second and subsequent recurrence, risk stratification follows a similar process; however, much less data is available. In the multicenter study discussed earlier, the predominant factor that contributed to improved survival for patients with second local recurrence was longer disease-free interval (HR 0.44) [50]. While no interval cut-off was established, the third quartile DFI was 32 months and the first quartile DFI was 8 months. Not surprisingly, patients deemed ineligible for surgery had worse OS, hazard ratio of 3.25, compared to those who underwent resection [50]. Interestingly, on multivariable analysis, histologic type and grade, receipt of chemotherapy, and receipt of radiation therapy were not significant factors for multiply recurrent disease. Additionally, this analysis highlights that many patients can still benefit from resection and that a proportion (24%) can even experience long DFI after surgery for second recurrence (median 57 months) [50]. Additional analyses corroborated that DFI > 12 months, and surgical resection were associated with improved survival [57].

## Clinical Decision-Making with a Focus on Recurrent Disease

The decision to move forward with a treatment (e.g. surgery) in RPS is nuanced beyond what current published data and objective risk stratification can offer. While we have described much of the “science” of medicine thus far, there is clearly the “art” that can at times be difficult to prescribe. This is in part the clinical judgement and experience of a sarcoma specialist and even more so, the collective input from a multidisciplinary team to determine the feasibility and benefit of each treatment modality in the context of others. Equally important is the patient’s realistic understanding of the proposed treatment, anticipated outcomes and potential downsides, including impact on quality of life. This underscores the high value of appropriate patient counseling, especially in recurrent RPS.

For both provider and patient, the goals of treatment should be recognized upfront, whether curative intent to durably eliminate disease or palliative intent to address a symptom or a combination of both. While in primary RPS, it is often straightforward to begin treatment with curative intent, in recurrent disease the formulation of goals and the path forward often become less clear. In locally recurrent RPS, and especially for LPS, the nihilistic view is that re-recurrence is nearly inevitable. In fact, especially in the United States, multiply recurrent disease is unfortunately quite common [29]. What should drive the decision-making to move forward with surgery? The factors that contribute to risk stratification should set the framework but opting for surgery is far from black and white and clinical judgement is essential. When the possibility of “cure” or prolonged DFS is unlikely or uncertain—based upon consideration of the tumor prognostic factors discussed above—it is critical to engaged in shared decision-making around treatments considered. In these settings, careful examination of patient and technical factors is paramount. For elderly patients or those with significant co-morbidities, factoring a patient’s overall fitness for surgery is crucial. Use of prognostic calculators such as ePrognosis [58]can aid in understanding overall prognosis for the patient independent of their cancer diagnosis. Given the complexity of reoperative surgery for RPS, often following prior multivisceral resection, careful consideration of the technical risks of the procedure is imperative. The American College of Surgeons National Surgical Quality Improvement Program Surgical Risk Calculator can be helpful in estimating risk of surgical morbidity and mortality [59, 60] with the caveat that it may be difficult to identify a procedural code that accurately reflects the potential procedure and that the calculator is not based purely on a cohort of patients with cancer. Lastly, when reoperation for recurrent RPS is considered with palliative intent, both surgeon and patient need to be clear about what symptom(s) are being targeted for palliation (e.g. intestinal obstruction, pain), the likelihood that the proposed procedure will successfully palliate the symptom, the presence of alternate, non-operative options for palliation, and the durability of the palliation provided by the operation [61]).

## Future Directions and Conclusions

Tumor molecular characterization has become ubiquitous as technology advances and costs decrease. Several studies have evaluated different molecular markers to assist in risk stratification. As an example, patients with RPS that had TERT alterations had worse DFS and OS [62] while a separate study identified that alterations in FOXO3 were associated with improved survival; alterations in the RECQL4, MN1, NOTCH1, and CNTRL genes identified patients with worse outcomes [63]. To improve prediction, gene panels have been utilized to dichotomize patients into low- and high-risk categories [64]. For STS, a 67-gene signature called the Complexity Index in Sarcoma (CINSARC) has been reported that predicts outcomes for patients [65]. CINSARC was evaluated alongside clinicopathological outcome prediction (Sarculator) to determine if this gene panel could enhance outcome prediction in RPS [66]. While CINSARC was predictive of DFS, it was not predictive of OS by itself. When it was added to Sarculator as a variable, however, it did not improve the discrimination for DFS or OS [66].

Evaluation of radiologic features to predict clinicopathological outcomes, radiomics, are also being examined [67]. Utilizing radiomics to perform additional risk stratification is provocative, as this is non-invasive and may be undertaken using existing imaging results (such as CT imaging). The largest study to date, Radiomics in Sarcoma of the Retroperitoneum (RADSARC-R), evaluated CT-based radiomic features of RPS [68]. This evaluation accurately identified tumor histologic type and grade with area under the receiver operator curve of 0.928 and 0.882, respectively [68]. These features that are predictive by radiomics are readily available through surgical specimen and core needle biopsy. Furthermore, utilizing the radiomic features in RADSARC-R, discrimination of DSS and OS were only marginally enhanced when utilized with Sarculator [67]. In the future, radiomics could generate an additional data point in the risk stratification of recurrent disease but it currently remains in its infancy.

Circulating biomarkers remain less established in STS, but there are ongoing trials evaluating the clinical utility of these markers (NCT03896620). Circulating tumor DNA (ctDNA) remains the most common circulating biomarker; others including exosomes and micro RNAs also exist and have been evaluated in RP LPS [69, 70]. Collectively, circulating biomarkers represents a unique tool that could aid in disease management on many levels including risk stratification (e.g., for early recurrence) and harbor the potential to refine selection of patients that may benefit from systemic therapies.

Overall, the field continues to progress forward in novel ways that will help risk stratify patients and provide them with appropriate treatment. As we progress, more information will become available, and the mounting challenge will be constructing a cohesive picture from an increasing array of potentially useful data points in each patient. Artificial intelligence could facilitate this by incorporating multiple risk stratification modalities to facilitate decision making in these complex situations [71].

## Key References

- Raut CP, Callegaro D, Miceli R, Barretta F, Rutkowski P, Blay JY, et al. Predicting Survival in Patients Undergoing Resection for Locally Recurrent Retroperitoneal Sarcoma: A Study and Novel Nomogram from TARPSWG. Clin Cancer Res. 2019;25(8):2664-71. doi: https://doi.org/10.1158/1078-0432.CCR-18-2700.
  - ◦ This reference is of outstanding importance because it identifies the key factors in risk stratification for recurrent retroperitoneal sarcoma.
- van Houdt WJ, Fiore M, Barretta F, Rutkowski P, Blay JY, Lahat G, et al. Patterns of recurrence and survival probability after second recurrence of retroperitoneal sarcoma: A study from TARPSWG. Cancer. 2020;126(22):4917-25. doi: https://doi.org/10.1002/cncr.33139.
  - ◦ This reference is of outstanding importance because it identifies the key factors in risk stratification and outcomes for patients with multiply recurrent retroperitoneal sarcoma.
- Tseng WW, Swallow CJ, Strauss DC, Bonvalot S, Rutkowski P, Ford SJ, et al. Management of Locally Recurrent Retroperitoneal Sarcoma in the Adult: An Updated Consensus Approach from the Transatlantic Australasian Retroperitoneal Sarcoma Working Group. Ann Surg Oncol. 2022;29(12):7335-48. doi: https://doi.org/10.1245/s10434-022-11864-y.
  - ◦ This reference is of importance because it represents a culmination of clinical outcomes and expert opinion in the nuanced management of recurrent retroperitoneal sarcoma.

## Data Availability

No datasets were generated or analysed during the current study.
